# Supplementary figures and images for: Runx1 shapes the chromatin landscape via a cascade of direct and indirect targets
Source: PLoS Genet. 2021 Jun 10;17(6):e1009574. doi: 10.1371/journal.pgen.1009574 (PMC8219162; doi:10.1371/journal.pgen.1009574)

# S1 Fig

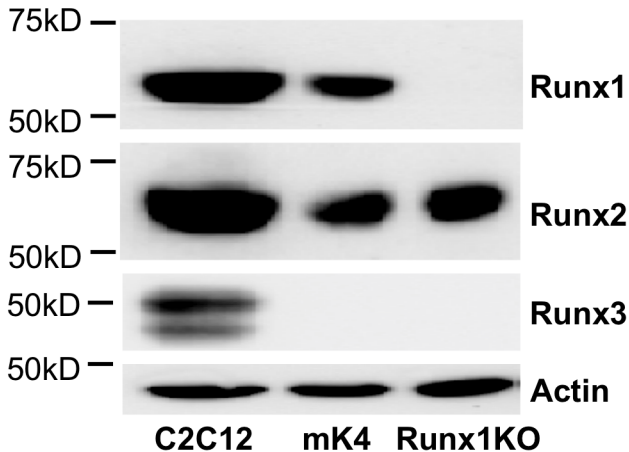

Supplement: S1 Fig — (PDF) [file pgen.1009574.s001.pdf]

S2 Fig

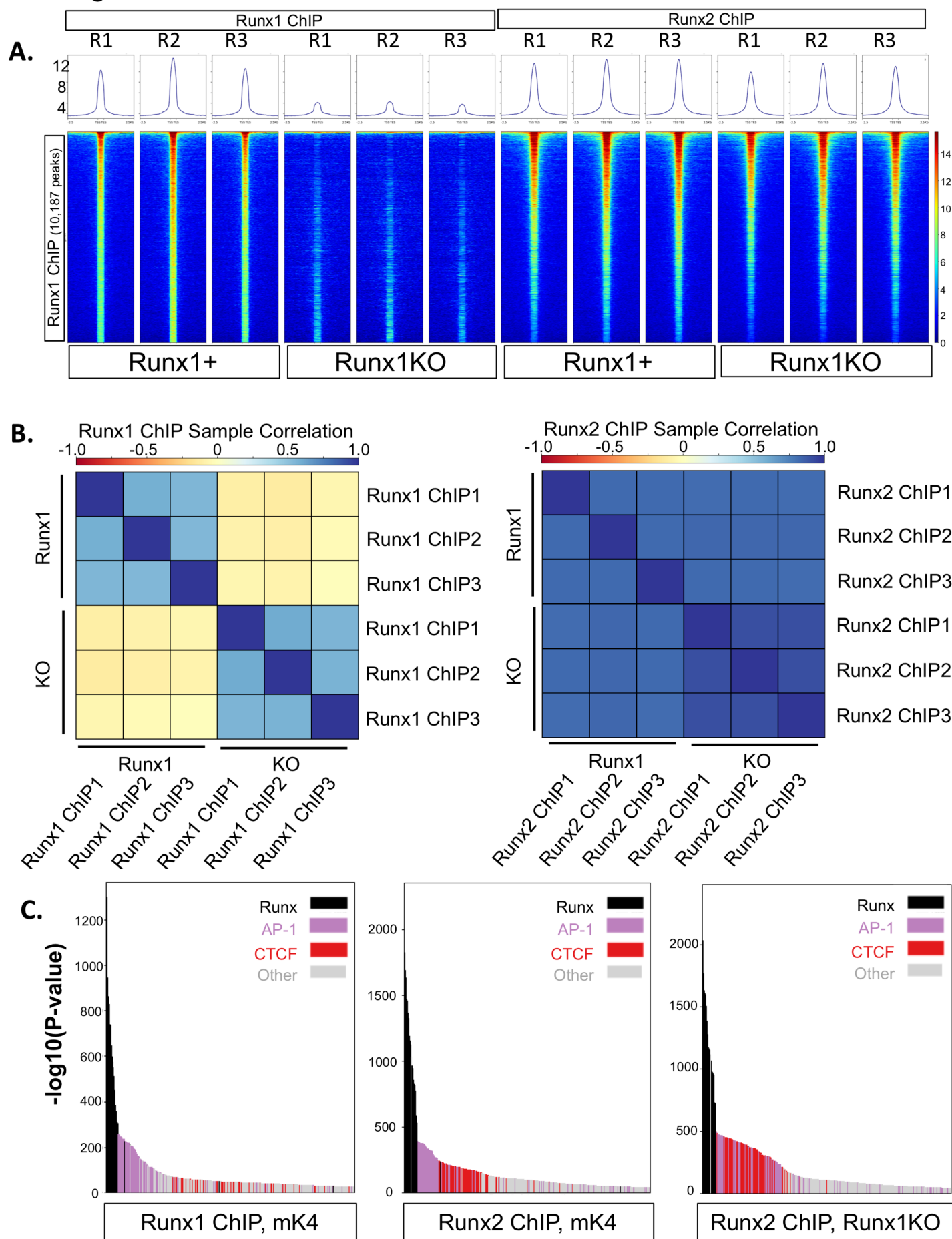

Supplement: S2 Fig — A) Heatmaps of Runx1 and Runx2 ChIP reads mapped to Runx1 ChIP peaks showing the reproducibility of the ChIP replicates. B) Correlation matrix for Runx1 (left) and Runx2 (right) ChIP showing strong correlation between samples C) Bar graphs of transcription factor motif enrichment -log10 p-values in the Runx1 ChIP in mK4 cells and Runx2 ChIP in mK4 and Runx1KO cells that confirm the strongest enrichment of Runx motifs. (PDF) [file pgen.1009574.s002.pdf]

**S3 Fig**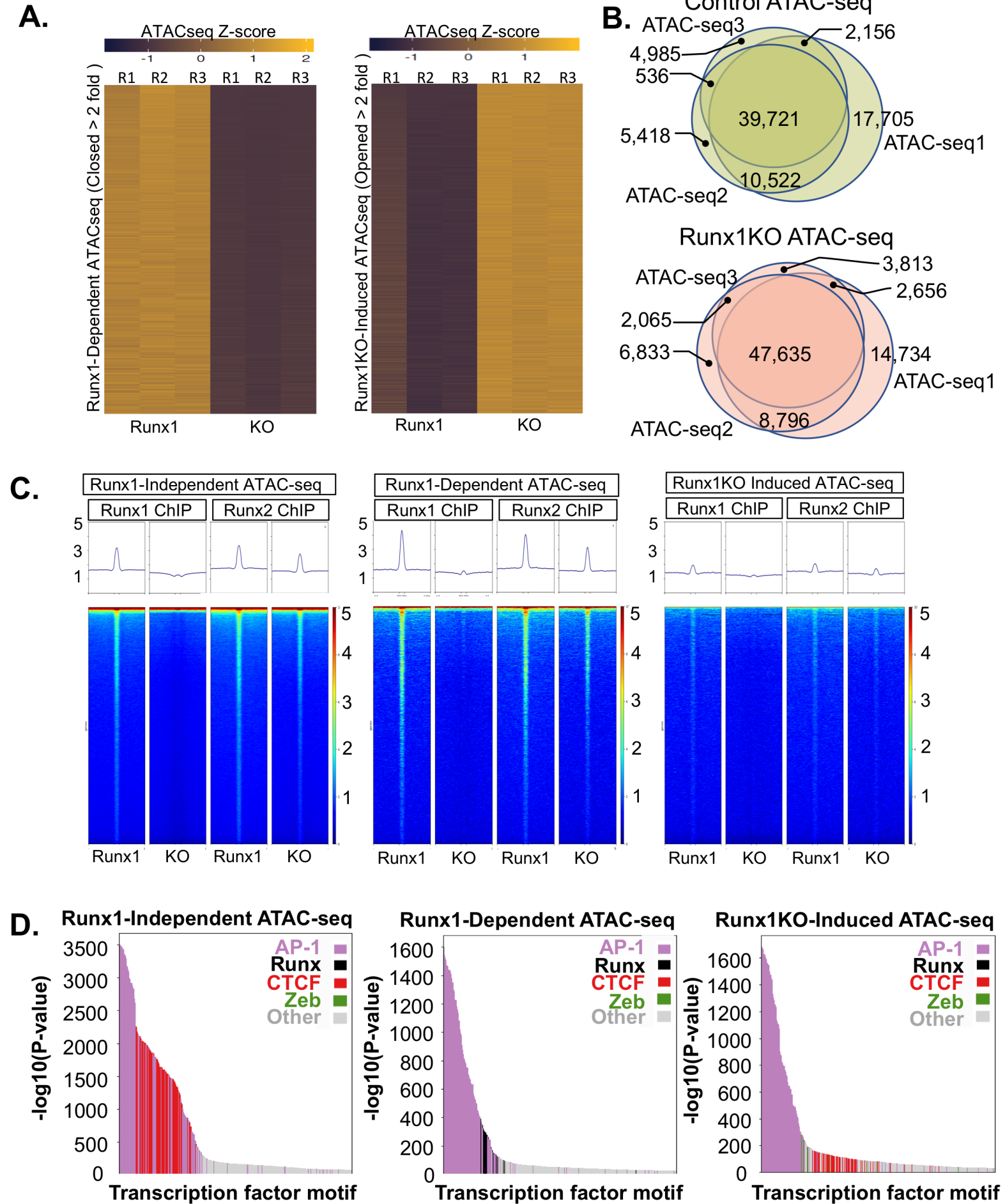

Supplement: S3 Fig — A) Heatmap of Z-scores of individual ATAC-seq samples from mK4 or Runx1KO cells in Runx1-dependent or Runx1KO-induced ATAC-seq peaks showing the reproducibility of the ATAC-seq signal in the replicates and the differences between the mK4 and Runx1KO cells. B) Venn diagrams displaying the overlap of the ATAC-seq peaks between replicates. C) Heatmaps of Runx1 or Runx2 ChIP showing Runx1 binding to both the Runx1-independent and Runx1-dependent ATAC-seq peaks but very little binding to the Runx1KO-induced ATAC-seq peaks. D) Bar graphs of -log10 p-values of motif enrichment in the 3 different classes of ATAC-seq peaks. Runx1-independent ATAC-seq peaks are enriched for AP-1 and Ctcf motifs, Runx1-dependent ATAC-seq enrich for AP-1 and Runx motifs, and Runx1KO-Induced ATAC-seq display enrichment of AP-1, Zeb, and Ctcf motifs. (PDF) [file pgen.1009574.s003.pdf]

**A.**

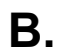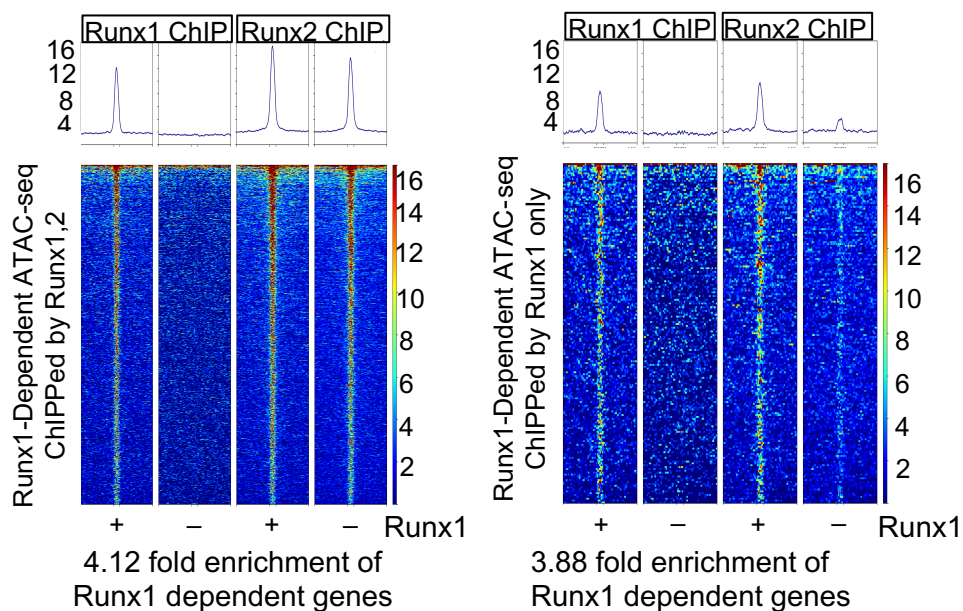

Supplement: S4 Fig — A) Genomic snapshots of Runx1 and Runx2 ChIP and ATAC-seq around the Runx1KO downregulated genes Twist2, Tnc, Zfp810, Inhbb, Lef1, and Igfbp4 that shows Runx1 and Runx2 binding to ATAC-seq peaks that are lost in Runx1KO cells. B) Heatmaps Runx1 or Runx2 ChIP on Runx1-dependent ATAC-seq peaks split into those sites that retain Runx2 binding in Runx1KO cells or sites where Runx2 binding is lost. The Runx1-dependent ATAC-seq that lose Runx2 binding in Runx1KO cells fail to enrich for genes that are down-regulated in Runx1KO cells more than the Runx1-dependent ATAC-seq that retain binding of Runx2. (PDF) [file pgen.1009574.s004.pdf]

S5 Fig

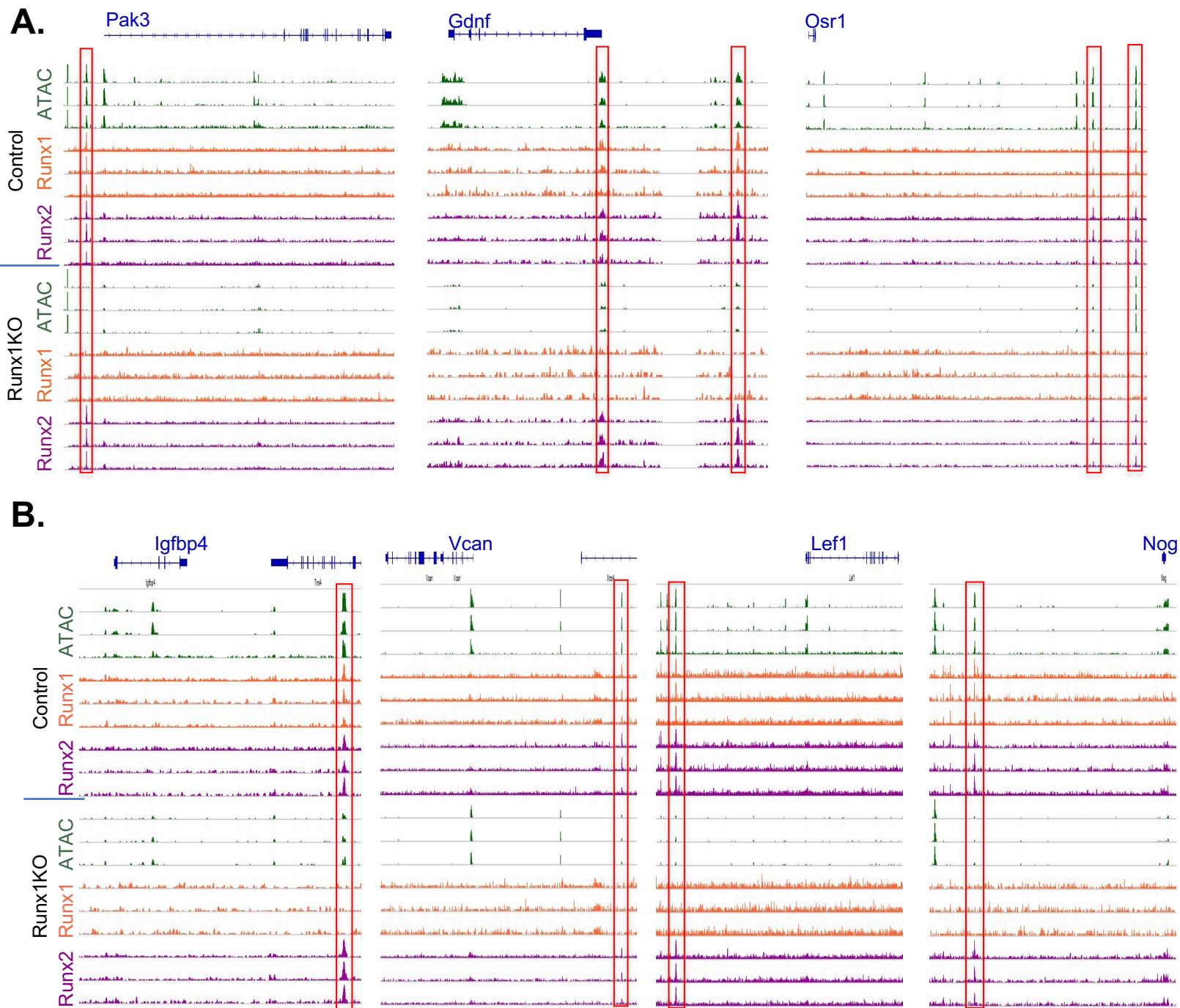

Supplement: S5 Fig — A) Genomic snapshots of aforementioned Runx1 target genes showing the reproducibility of the signal in all three of the replicates for the ATAC-seq, Runx1-ChIP, and Runx2-ChIP. B) Additional examples not discussed in the text. (PDF) [file pgen.1009574.s005.pdf]

S6 Fig

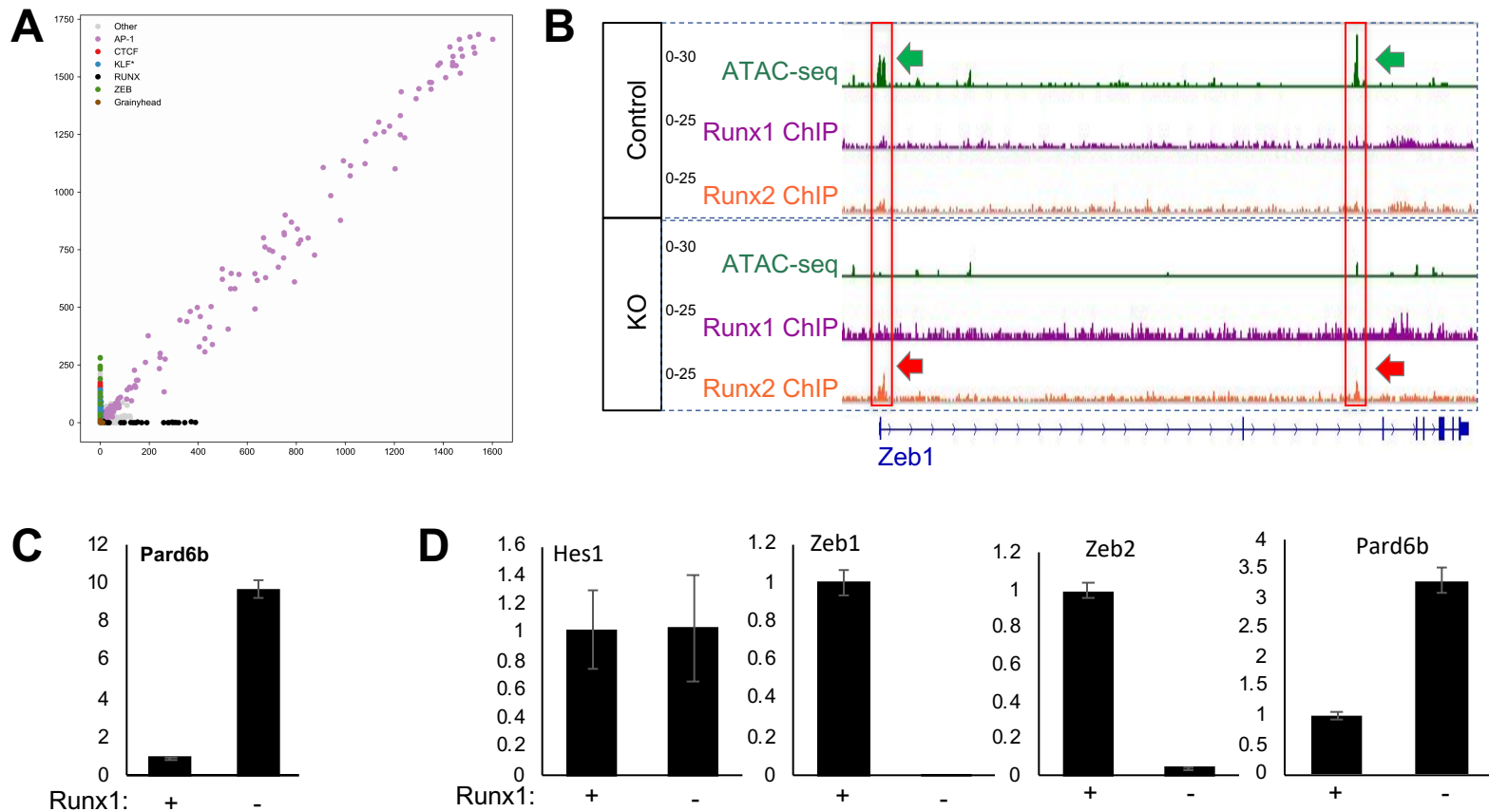

Supplement: S6 Fig — A) Plot showing transcription factor motif enrichment (-log10 p-value) in Runx1-dependent versus Runx1KO-induced ATAC-seq peaks that shows AP-1 motifs are strongly enriched in both but Zeb, Ctcf, Klf, and Grainyhead motifs are specific to Runx1KO-induced ATAC-seq peaks while Runx1 motifs are enriched only in Runx1-dependent ATAC-seq peaks. B) Genomic snapshot of Zeb1 that shows the loss of ATAC-seq signal at the TSS that is lost in Runx1KO cells, which is consistent with the loss of expression in these cells. C) Graph of normalized RNA-seq reads showing the upregulation of Pard6b in Runx1KO cells as expected for a Zeb repressed gene. D) RT-qPCR analysis confirming the dramatic down-regulation of both Zeb1 and Zeb2 and upregulation of Pard6b in Runx1KO cells, but lack of difference for the unrelated gene Hes1. (PDF) [file pgen.1009574.s006.pdf]

S7 Fig

A

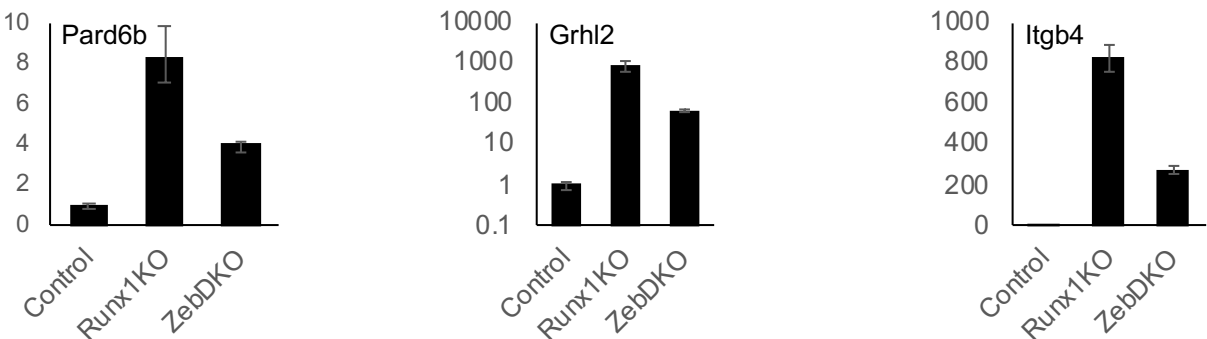

B

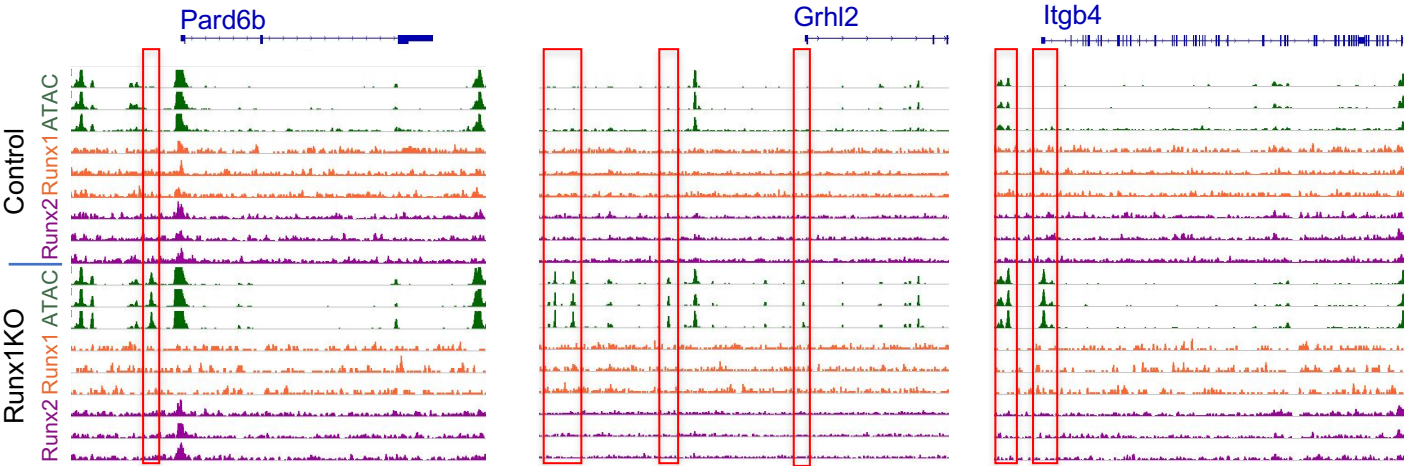

C

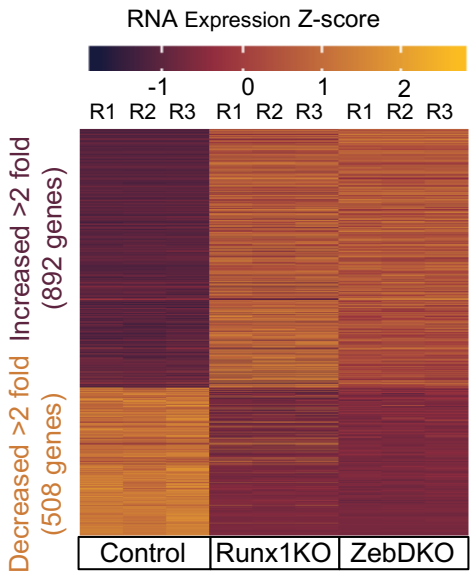

D

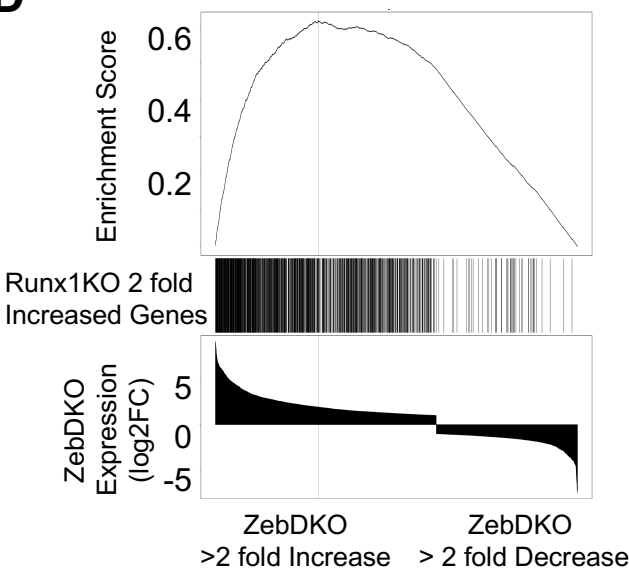

Supplement: S7 Fig — A) RT-qPCR showing that Runx1KO and ZebDKO cells have similar upregulation of Pard6b, Grhl2, and Itgb4. B) Genomic snapshots of Pard6b, Grhl2, and Itgb4 displaying chromatin opening of potential enhancers containing Zeb motifs in all three ATAC-seq replicates (red boxes) from Runx1KO cells. C) Heatmap of genes displaying over 2 fold change in expression in Runx1KO and ZebDKO cells compared to control mK4 cells. D) GSEA analysis showing enrichment for genes that were increased over 2 fold in Runx1KO cells in the set of genes that were increased by over 2 fold in the ZebDKO cells, as expected given the known repressive function of Zeb proteins. (PDF) [file pgen.1009574.s007.pdf]
