# Supplementary material for: Runx1 shapes the chromatin landscape via a cascade of direct and indirect targets
Source: PLoS Genet. 2021 Jun 10;17(6):e1009574. doi: 10.1371/journal.pgen.1009574 (PMC8219162; doi:10.1371/journal.pgen.1009574)
Supplement: Attachment — Submitted filename: Hass et al_ReviewerComments.docx [file pgen.1009574.s014.docx]

The authors wish to thank all the reviewer for their careful reading of manuscript and for raising important points, helping to improve the manuscript. Our point-by-point response is provided below. When needed, reviewer-only figures were added.

**Reviewer #1**

The manuscript reports the contribution of the Runx1 transcription factor to chromatin organization that is functionally linked to control of gene expression. Utilizing CRISPR-ablation of Runx1, the consequences for chromatin accessibility, evaluated by ATAC seq, was examined and related to gene expression that was assessed by RNA-seq. The observed modifications in gene expression are consistent with well-established parameters of biological control and pathology that have been reported for Runx transcription factors. The influence on the Runx1 mediated genomic/epigenomic landscape was directly addressed by Chip seq. The shared and unique activities of Runx1 and Runx2 were examined with insight into Runx control of proximal and distal genomic regulatory domains. The influence of Runx1 on Zeb1 and Zeb2 expression, within the context of chromatin accessibility was reinforced by overexpression experiments. And, while somewhat un-physiological, these experiments provided insight into regulatory cascades that are linked to Runx regulation of the genomic landscape. The experimental approaches were straightforward and findings were appropriately discussed in relation to results in the literature, particularly those secured from studies on Runx1 control in mammary epithelial and breast cancer cells. The reported findings are a valuable contribution to advancing understanding of Runx1 control of the chromatin landscape and competency for gene expression. The results provide a blueprint for mechanistically exploring Runx regulation of epigenetic control and chromatin organization that supports transcriptional competency.

**Reviewer #2**

The transcription factor RUNX1 plays important roles in regulating gene expression in many cell types, including hematopoietic, skin, and bone cells. It appears to have positive effects on some genes and repressive effects on other. This manuscript delves more deeply into positive and negative roles of RUNX1 in the mouse kidney cell line mK4, which is derived from metanephric mesenchymal cells. This cell line can be considered an informative model for cells undergoing the mesenchymal to epithelial transition, a process important in development of the kidney. The role of RUNX1 in kidney development has not been extensively studied. The authors generated a RUNX1 knockout (KO) subline of mK4 cells, and determined the impact of RUNX1 loss on the transcriptomes by RNA-seq, the genome-wide binding profiles for RUNX1 and RUNX2 by ChIP-seq, and chromatin accessibility by ATAC-seq. All these determinations were in triplicate to allow stringent determinations of changes. The results show that primary role of RUNX1 in this kidney cell context is that of a transcriptional activator. Most of the genes whose gene expression is positively regulated by RUNX1 (down-regulated in the KO) have, within a reasonable vicinity, plausible candidate enhancers bound by RUNX1. In contrast, most of the genes whose expression is negatively regulated by RUNX1 (up-regulated in the KO) do not appear to be direct targets of RUNX1. Instead, studies of motif enrichment and further biochemical examination reveal a role for ZEB1 and ZEB2 as repressors. The authors conclude that the role of RUNX1 is to activate expression of the repressor proteins, thereby leading to gene repression by an indirect mechanism. These experiments and analyses lead to important new insights into the mechanisms of regulation by RUNX1. The authors also provide some discussion about the possible application of these insights into RUNX1 and ZEB1 and 2 function in other cellular contexts.

The experiments are performed carefully and with a good replication structure. The analyses are appropriate and rigorous. The text is clear for the most part but it could be more concise.

The manuscript could be improved by addressing the following specific points.

(1) The results of the triplicate determinations are shown for RNA-seq in Fig. 2, but the patterns across replicates are only shown in the heatmaps of signal density in peaks in the Supplement. Showing at least some of the differentially bound or accessible regions (e.g. those in Fig. 3 F and G) for all three determinations would provide compelling examples of consistency in the data. Such a figure panel would be a strong addition to the Supplementary figures.

We thank the reviewer for this suggestion. We included genomic snapshots showing all 3 of the replicates for the ATAC-seq and ChIP datasets as a Supplemental Figure 3G, H.

(2) The authors should state that mK4 cell line is from mouse early in the paper; the first I read of mouse as the species of origin was in the Discussion.

We have changed the text to now say “mouse metanephric mesenchyme-derived mK4 cells” when the cells are first mentioned in the abstract.

(3) Some important labels are obscured in "Figure 2-Supplemental Figure 1", e.g. which panels for RUNX1 ChIP-seq and which are for RUNX2?

We have revised the figure and added Runx1 ChIP and Runx2 ChIP headers. The figure was renamed in the revised manuscript as Supplemental Fig 2.

(4) What is the x-axis in Fig. 2C and 3C and in "Figure 2-Supplemental Figure 1B"? The label says transcription factor binding motif, but there are multiple values for each of the motifs in the color key. Are there many motifs in the RUNX class, in the AP1 class, etc?

Yes, there are numerous motifs available for most transcription factors (from different technologies and sources). We measured relative enrichment for each motif, and each dot represents a unique motif. In Supplemental Table 3, the reader can filter for RUNX transcription factor motifs and assess enrichment across different motifs, for example the Runx motifs (M01307: NWAACCGCAA and M09633: SAAACCACAG). We clarified this in the text as follows:

“HOMER transcription factor binding site motif enrichment analysis using mouse motifs from the Cis-BP database including all reported motifs for various TFs (25) identified Runx motifs as the most highly enriched in the control cell dataset (p-value 1 x 10^-1298^) (Fig 2C and Supplemental Table 3).”

(5) Lines 203-205: The conclusion drawn about direct repression is overly broad. The results presented do not make a case for one conclusion that applies to all repressed genes. Just using the numbers in the preceding sentence, it seems that the data DO support a model for a direct role of RUNX1 in repression of about 30% of the genes whose expression is up-regulated in the Runx1 KO. Of course, that leaves a majority of the upregulated genes as not consistent with a direct role of RUNX1, and that would be an appropriate way to describe the conclusion.

The 30% of upregulated genes having a Runx ChIP peak in the vicinity is just barely above what would be expected by chance alone suggesting that most of these may be landing near an upregulated gene due to chance alone. Nevertheless, we have softened our argument to suggest that some of these sites may represent locations of Runx1 repression.

“While the limited overlap of Runx1 ChIP peaks near upregulated genes cannot exclude the possibility that some of these may represent sites of Runx1 mediated repression, the results are consistent with the loss of expression in Runx1KO cells of direct Runx1 targets and less consistent with a model in which upregulated genes were repressed directly by Runx1.”

(6) In Fig. 2B, are the genomic regions with the RUNX2 ChIP-seq signal the same ones and in the same order as those shown for the RUNX1 ChIP-seq signals? Or are they for the peaks of RUNX2 binding, ordered by the strength of RUNX2 signal?

Fig 2B shows the Runx2 ChIP-seq signal on the Runx1 ChIP peaks in the same order as the Runx1 ChIP-seq signal. We have now clarified this issue in the figure legend.

These heatmaps indicate a substantial overlap in binding between RUNX1 and RUNX2, but the Venn diagram in Fig. 2D indicates that roughly half the peaks overlap. These panels need to be reconciled.

In some cases, we obtained ChIP-seq reads that were not called as peaks by MACS2. The lower overlap of called peaks in the Venn diagram is likely due to the Runx2 signal being just below the threshold for peak calling for many of the peaks on the lower half of the heatmap even though we agree there is evidence of co-binding. Furthermore, the RELI analysis confirmed the very significant overlap of the Runx1 and Runx2 ChIP (138.5 fold enrichment, corrected p-value 1.45 x 10^-218^). We added this clarification to the legend of Figure 2.

(7) The observations on lines 257-258 should be expanded. What fraction of the RUNX1-dependent ATAC-seq peaks are also bound by RUNX1? Is it a high majority? More information is presented later, but the relevant supplemental figure panels are missing, and no explicit answer is given to this question.

Around 10% of Runx1 ChIP peaks overlap with Runx1-dependent ATAC-seq, whereas only 1% of Runx1 ChIP peaks overlap Runx1KO-induced ATAC-seq peaks. The Runx1 ChIP signal in the Runx1-dependent ATAC-seq peaks in the heatmaps, which are now included, shows that most Runx1-induced ATAC-seq have Runx1 ChIP signal and suggests that Runx1 binding may be occurring at most of these sites but is subthreshold for the region to be called a Runx1 ChIP peak.

“The 2nd most enriched motif class in Runx1-dependent open chromatin regions was Runx (p-value 1 x 10^-388^) and as expected, the dataset had highly significant overlap with our Runx1 ChIP data (26.43 fold enriched, Bonferroni corrected p-value 2.16 x 10^-211^; Supplemental Table 4). This enrichment of Runx1 ChIP peaks within Runx1-dependent ATAC-seq peaks was nearly 10 fold higher than the enrichment of Runx1 ChIP peaks within Runx1KO-induced ATAC-seq peaks (5.2 fold enrichment, corrected p-value 9.1 x 10^-67^), consistent with a role for Runx1 in promoting and maintaining open chromatin in these cells.”

Supplemental Fig 3C middle panel:

(8) "Figure 3-Supplemental Figure 1" appears to be missing panels A, B, and C.

The Supplemental Fig 3 panels A, B, and C were mistakenly lost during conversion to PDF and this oversight has now been fixed. They are now panels A, C, and D due to the addition of a new panel B.

(9) For GSEA (Fig. 3D), the gene sets examined need to be stated in the text or figure legend and labeled in the Figure. What is blue and what is red along the x-axis?

We have added labels to the figure to clarify and described in the legend.

(10) line 280: What exactly is being referred to by the term "pioneer/activator function"? The concept of a pioneer function has evolved, and in some contexts it refers to an activity that opens previously inaccessible chromatin. Is that what is meant, and if so, how is that involved in maintenance of accessibility?

We have removed references to pioneer activity from the text. Our data do not specifically show that Runx1 is responsible for displacing nucleosomes on these regions so the manuscript has been changed to refer to Runx1 activity as maintaining chromatin accessibility throughout, which is better fitting for the data that we provide.

(11) lines 288-290: What aspect of the data in Fig. 3E show a "slightly decreased" RUNX2 ChIP signal at the RUNX1-dependent ATAC-seq sites in Runx1KO cells? The heat maps look the same for + and - RUNX1. The specific examples are pretty clear in panel F, but the overall pattern in panel E does not show a clear decrease.

We agree the decrease in Runx2 binding at the Runx1-dependent ATAC-seq sites in the heatmap is subtle and appears to only occur at a minority of sites. We have removed this sentence from the manuscript.

(12) line 395: need to add "those encoding" before "several other transcription factors".

We modified the text as suggested.

(13) lines 411-412: What data in this paper support the conclusion that RUNX1 appears to "act by making chromatin accessible to other TFs"? In order to see such an effect, one might expect an analysis of DNA sites that were previously in inaccessible chromatin, but which become accessible upon introduction of RUNX1. It is not clear how a knock-out experiment shows an effect on initiation of chromatin opening. The loss of accessibility upon RUNX1 knock out supports a role in maintaining accessibility, as the authors point out in other text.

We agree that our data are unable to discriminate between a pioneer function of opening of closed chromatin versus the related function of being required for the maintenance of chromatin opened by a separate pioneer factor. Thus, we have changed all mentions of pioneer activity of Runx1 to simply refer to maintenance of chromatin accessibility, which is better supported by the data we provide. We changed the test to read:

‘In mK4 cells, Runx1 appears to largely act by maintaining chromatin accessible to other TFs, and by maintaining the expression of Zeb repressors to prevent a myriad of other sites from responding to their regulators”.

**Reviewer #3**

The article by Hass et al. describes how knocking out RUNX1 in a kidney derived cell line leads to changes in chromatin organisation and gene expression even at genes that are also bound by RUNX2. They also demonstrate that lost accessible sites are enriched in AP-1 motif. In addition, they show that genes which are down-regulated are direct Runx1 targets but genes which are up-regulated are not. They propose that the up-regulation of such genes is caused by the down-regulation of ZEB repressor proteins as sites associated with such genes are enriched in ZEB motifs.

The data is of good quality and the results are interesting and if taken further, could expand upon known functions of RUNX1 in a novel cellular context.

However, I am afraid that this study is not developed far enough to support the conclusion put forward by the authors, this holds true in particular for the proposed role of ZEB proteins

Major Comments

1. Loading controls (such as Gapdh or B-actin) must be shown for western blots in Figure 1, Figure 4 and Supplementary Figure 1.

We have repeated all the Western blots to include β-actin loading controls.

2. Lines 183-185 and 252-255 presence of an AP-1 motif alone is not sufficient to describe these cis-regulatory regions as enhancers. Along the same vein: The loss of AP-1 motifs does not mean that this factor is binding there. To ensure that RUNX1 is required for AP-1 binding, ChIP data need to be provided

We acknowledge that simply containing AP-1 sites would not be sufficient to definitively call a region an enhancer. However, these are open chromatin regions that were enriched for AP-1 sites and we referred to them as “**likely** **enhancers**” in both instances. There is substantial evidence in the literature that AP-1 sites are a general indicator of enhancers in addition to the reference that we provided. For example, Vierbuchen et al (PMID 29272704), found that AP-1 sites allow enhancer selection by cell-type specific TFs; Kwasnieski et al (PMID 25035418), showed that AP-1 sites were the “most significantly enriched motif in highly expressed CREs”; and Biddie et al (PMID 21726817), reported AP-1 opening of enhancers and AP-1 binding was required for other TF to regulate accessibility. We have now included these references in the text. While investigating the role of Runx1 in regulating AP-1 binding is an interesting avenue of investigation, it is beyond the scope of this paper and not a central point of this manuscript and has been previously investigated in Pencovich et al., 2011 (PMID 20959602). Nevertheless, our enrichment analysis (RELI) showed that the Runx1 ChIP peaks displayed very significant overlap with FOSL1 ChIP-seq peaks (corrected p-value of 8.79 x 10^-210^) in C2C12 cells and various Jun proteins in CH12 cells, suggesting that AP-1 complexes do bind these regions. Additionally, the Runx1 ChIP peaks also significantly overlapped with multiple enhancer marks such as H3K27acetylation or H3K4dimethyl or trimethyl in multiple cell types, further suggesting that these regions are likely enhancers.

Additionally, we have now performed H3K27 acetylation ChIP in control and Runx1KO cells and found that Runx1 and Runx2 ChIP significantly overlapped with H3K27 acetylation that was enriched in the control cells compared to Runx1KO (Runx1 enrichment 33.36 fold, p-value 2.17 x 10^-182^; Runx2 ChIP 36.75 fold, p-value 5.49 x 10^-203^). Furthermore, when we filter our Runx1 or Runx2 ChIP to those that overlap predicted AP-1 binding sites, we still observe strong enrichment for H3K27acetylation specific to control cells for both Runx1 and Runx2 ChIP (Runx1 enrichment 35.48 fold, p-value 2.83 x 10^-180^; Runx2 ChIP 38.60 fold, p-value 7.63 x 10^-200^). In contrast, Runx1 and Runx2 ChIP peaks that did not overlap with predicted AP-1 binding sites failed to significantly enrich for the H3K27 acetylation in control cells, suggesting that the presence of AP-1 binding is likely necessary for H3K27 acetylation to occur. A similar analysis of our Runx1-dependent ATAC-seq open chromatin found that they also significantly enriched for H3K27 acetylation specific to control cells (24.2 fold enriched, p-value 1.01 x 10^-171^). Overall, these new analyses confirm that the genomic sites that we have identified are likely bound by AP-1 to function as enhancers, given their significant overlap with H3K27 acetylation signal.

We have added discussion of these points to the paper as indicated below:

“Notably, enrichment analysis revealed that the Runx1 ChIP regions overlapped very significantly with FOSL1 and various Jun proteins in other cell types, consistent with the observed AP-1 motif enrichment in our ChIP datasets and supporting the hypothesis that Runx1 binds to enhancers. Accordingly, the Runx1 ChIP peaks enriched significantly for enhancer marks such as H3K27 acetylation, H3K4 methylation, H3K4 dimethylation, H3K4 trimethylation, and DNase sensitivity in multiple cell types, strengthening the assertion that the Runx1 bound regions are likely enhancers. We confirmed these associations by performing H3K27 acetylation ChIP-seq in control and Runx1KO cells, which revealed that Runx1 ChIP-seq peaks significantly overlap with H3K27 acetylation peaks that were stronger in control cells compared to Runx1KO cells (33.36 fold, p-value 2.17 x 10-182) (Supplemental Table 4). Filtering Runx1 ChIP peaks only to those that overlapped with predicted AP-1 binding showed similar enrichment for H3K27 acetylation in control cells (Runx1 enrichment 35.48 fold, p-value 2.83 x 10-180). By contrast, Runx1 ChIP peaks that did not overlap with predicted AP-1 binding sites were not significantly enriched for control cell specific H3K27acetylation.”

3. The fact that some down-regulated genes cannot be rescued by the presence of RUNX2 is very interesting, but again, the mechanistic basis of this finding is unclear. How does histone modification look like at these regions? Could chimeras between RUNX1 and RUNX2 be used to examine which domain of RUNX1 is required for the activation of these genes?

Our RELI analysis showed significant overlap of the Runx1-dependent ATAC-seq regions with multiple different histone modifications (H3K27 acetylation, H3K4 methylation, H3K4 dimethylation, and H3K4 trimethylation), and DNase sensitivity in several distinct cell types, which suggests that these are likely enhancers used by many different kinds of cells (Supplemental Table 4). As mentioned above, we performed H3K27acetylation ChIP in the control and Runx1KO cells and interestingly the Runx1KO display a reduced but not abolished H3K27acetylation of the Runx1-dependent ATAC-seq regions, while there was essentially no difference at the Runx1-independent ATAC-seq regions. RELI analysis on the new H3K27acetylation data showed that the Runx1-dependent ATAC-seq regions overlapped significantly with H3K27acetylation enriched specifically in control cells compared to the Runx1KO cells (24.2 fold, p-value 1.01 x 10^-171^). We have provided a snapshot of the Runx1 target gene Lef1 that shows the Runx1 and Runx2 bound enhancer that closes (by ATAC-seq) in the Runx1KO cells and has reduced, but not abolished H3K27 acetylation signal. We have also included a track of FOSL1 ChIP from C2C12 cells (which shows significant overlap with the Runx1 ChIP by RELI analysis) as further visual evidence that AP-1 complexes are able to bind to these regions, at least in other cells.

Unfortunately, mK4 cells do not survive over-expression of Runx1 so the chimera analysis can not be performed in these cells. However, we have found that Runx1 deletion in C2C12 cells causes a clear strong phenotype despite the presence of both Runx2 and Runx3 and we are able to rescue these cells with Runx1 plasmids. We are in the process of performing the Runx1 and Runx2 chimera analysis in the C2C12 cells, but this is separate project that will be reported elsewhere.

4. Figure 2/Supplementary Figure 2 the Venn diagram in panel D shows around a quarter of Runx2 peaks are each gained and lost following Runx1 KO. The authors mention that replicates were well correlated but this is not easy to confirm with the heatmaps shown. Here either a correlation matrix should be provided or a similar Venn diagram of peak numbers to see if the changes in response to Runx1 KO are more than expected by chance/technical variation.

We now provide correlation matrix for the Runx1 and Runx2 ChIP experiments that shows that the replicates are highly correlated and lack of correlation of the Runx1 ChIP in the Runx1KO cells, as expected. These are included in the manuscript as Supplemental Fig 2B.

5. It is puzzling to see that RUNX2 binding can be associated with inaccessible chromatin - how many of its peaks lie within open chromatin (related also to Figure 3) and what distinguishes these from the inaccessible sites?

Approximately, 42% of Runx2 ChIP peaks are located in closed chromatin and 58% are in open chromatin. We have provided heatmaps of the Runx2 ChIP regions that are present in either open chromatin or closed chromatin that show that Runx2 ChIP does indeed occur in regions that are shown to be closed by ATAC-seq, although the peak height is slightly less than in open chromatin regions. Additionally, we compared TF motif enrichment between Runx2 ChIP in open vs closed chromatin and both types showed similar strong enrichment of Runx motifs, confirming that both open and closed sites are likely directly bound by Runx2 (see figure below). The Runx2 ChIP in open chromatin specifically enriched for AP-1 motifs (see figure below), which fits with these regions being potential open active enhancers. Conversely, the Runx2 ChIP in closed chromatin was enriched for CTCF sites (see figure below), which is consistent with CTCF being involved in closing and silencing these regions. Interestingly, Runx2 and CTCF have previously been shown to frequently co-occupy genomic regions in MC3T3 cells, which also reported that 37% of Runx2 ChIP peaks in matrix depositing osteoblasts were in closed chromatin as defined as being located outside of DNase Hypersensitivity Sites (Tai et al., 2017 (PMID 29176792). Thus, the binding of Runx2 to closed chromatin has been observed elsewhere and seems tangential to the Runx1 focus of this manuscript, so we have not incorporated this information into the manuscript but provide it below for the reviewer.

6. Results/discussion: Lines 203-205, Figure 4. A central claim to the article is that RUNX1 is not acting as a repressor via Zeb proteins. However, the data does not support this claim. 30% of upregulated genes having a nearby RUNX1 peak still seems high even compared to the 45% of downregulated genes although a p-value is only given for downregulated genes - is this because it is also higher than expected by chance? In the absence of Hi-C data, how do they know which binding site belongs to which gene. It is known that only about 50% of all ChIP sites belong to the nearest promoter – which will create a problem with the statistical significance.

We argue that 30% vs. 45% is a huge difference, given the genome-wide scale. Further, there are exceptions to practically any “rule” in biology. Certainly, RUNX1 will be located within repressive elements at some loci – we simply make the claim that direct repression is not its main role in this mK4 context.

We agree that Hi-C (or related) looping data would be useful for assigning peaks to gene targets. However, generation of these data is well beyond the scope of this study. While imperfect, many (likely most) genomics studies still use gene proximity as a proxy for assigning gene targets. Even though this approach may not be sufficient to assign every enhancer to the gene it regulates, our statistical analysis was sufficiently powerful enough to clearly detect enrichment for genes that lost expression in Runx1KO cells by nearest gene annotation of enhancers that were ChIPPed by Runx1 and lost accessibility in the Runx1KO ATAC analysis.

7. In the follow up experiments binding at these genes by Zeb1/2 is not documented by ChIP so there is no direct evidence that these proteins are acting as the repressors at these sites. The authors make a point that there is no antibody available, but they work with a cell line into which tagged ZEB proteins could be introduced and expressed in an inducible fashion, which would nail the issue.

To address this concern, we have generated Zeb1 and Zeb2 (ZebDKO) double knockout cells and confirmed that they exhibit significant upregulation of *Grhl2*, *Pard6b* and several other targets by Q-PCR. This supports that loss of Zeb expression in Runx1KO cells is likely responsible for the upregulation of these genes. Additionally, we performed RNA-seq on the ZebDKO cells and found that the gene expression changes correlated very strongly with those observed in the Runx1KO cells, with the strongest correlation being observed in upregulated genes in both Runx1KO and ZebDKO cells, as would be expected given the role of Zeb proteins in repression.

Further, the putative enhancers near *Grhl2* that were open specifically in Runx1KO cells and contained Zeb motifs are now shown to be open by ATAC Q-PCR in the ZebDKO cells compared to control cells. These ZebDKO data have been inserted as a new Fig 5. The GSEA analysis of genes that are upregulated in both cellular backgrounds is available in Supplemental 5C. A heatmap showing the large number of genes with similar changes in expression in both knockout cell lines is provided in Supplemental Fig 5D.

8. Could RUNX1 and ZEB proteins be acting in a complex and therefore Runx1 binding is indirect given the lack of RUNX motifs? Is the t-test in Fig4 panels G & I vs the Runx1KO? If so this needs to be done against the control too to confirm if there is a full rescue - there does not appear to be, further suggesting it is not ZEB proteins alone.

We performed co-immunoprecipitation experiments to check for a potential complex between Runx1 and Zeb1 and found no evidence for an interaction between these proteins under conditions in which Runx1 was able to efficiently co-immunoprecipitate its coactivator CBFβ. Given the lack of interaction between Zeb1 and Runx1, we do not discuss this possibility of a Zeb1/Runx1 complex in the manuscript but could mention this lack of direct interaction and provide the Western blots from this experiment as supplemental information if deemed necessary.

We agree that the Zeb2 expression did not lead to complete reversal of the Runx1KO upregulation of targets. The difference between the control (not expressing *Pard6*) and Runx1KO+Zeb2 for *Pard6b* was not significant (p-value =0.172) while there was a significant difference between them for *Grhl2* (p-value = 0.016). We now mention in the text that additional pathways may play a role in the Runx1KO cells.

“Because *Pard6b* expression levels did not fully return to control levels, there may be additional proteins contributing to its regulation. Alternatively, stable expression of Zeb might be required over a longer period.”

Additionally, we have now generated Zeb1/2 double-knockout (ZebDKO) cells and observed that many of the same targets upregulated in Runx1KO cells are similarly induced, such as Pard6b and Grhl2. Furthermore, we have generated ATAC PCR data showing that the identified regions near Grhl2 containing Zeb motifs that are opened in Runx1KO cells display significant opening in the ZebDKO cells, as well.

“To directly test whether loss of Zeb expression is responsible for the opening of chromatin and gene upregulation observed in Runx1KO cells, we again utilized CRISPR-Cas9 to knockout both Zeb1 and Zeb2 to generate ZebDKO cells. We successfully obtained several clones that deleted the exons containing the start codon for each (Fig 5B) and confirmed the loss of Zeb1 protein (Fig 5C). Global expression analysis by RNA-seq confirmed that much of the transcriptional deregulation observed in Runx1KO cells was recapitulated in ZebDKO cells, as shown in a scatter plot comparing the differentially expressed genes (DEG) in Runx1KO vs. control mK4 cells with those in ZebDKO vs. control mK4 cells (Fig 5E). These results are further illustrated in a heatmap of the genes displaying over 2 fold change in expression levels in the replicates comparing the control cells to the Runx1KO and ZebDKO cells (Supplemental Fig 5C). Gene Set Enrichment Analysis (GSEA) showed enrichment of genes upregulated in Runx1KO cells among genes significantly increased in ZebDKO cells (Supplementary Fig 5D). Specifically, we confirmed that *Grhl2*, *Pard6b* and *Itgb4* were all upregulated in Runx1-expressing ZebDKO cells by RT-qPCR (Fig 5D and Supplementary Figs 5A and 5B). The enrichment of Grhl motifs in the Runx1KO-induced ATAC-seq and the upregulation of Grhl2 targets (Ovol1, Cldn4, and Cgn) in both Runx1KO and ZebDKO RNA-seq datasets (Supplemental Table 1) suggest that *Grhl2* derepression has important functional consequences *in vitro.* Moreover, *Grhl2* repression by Zeb plays a critical role in reciprocal negative feedback loops between these factors during epithelial-mesenchymal transition (EMT) (39). These observations prompted us to analyze the regulatory region of *Grlh2* as a representative Zeb target. To determine whether the transcriptional upregulation in ZebDKO cells was due to opening of chromatin near this DEG, we performed ATAC in ZebDKO cells and used QPCR to compare the enrichment of several putative enhancers near the *Grhl2* locus (-40kb, -69kb, and -74kb) to their status in the control mK4 cells. The accessible areas gained in Runx1KO cells were also more accessible in the ZebDKO cells relative to control. By contrast, constitutively open (-32kb) or inaccessible (-36kb) regions were indistinguishable between the control and ZebDKO cells (Fig 5G). Combined, Zeb knockout in Runx1-expressing cells and overexpression in Runx1KO cells demonstrate that Zeb proteins are both required and sufficient to suppress a cohort of targets upregulated in Runx1KO cells, and the loss of Runx1 unleashed a TF cascade due to the derepression of Zeb. Thus, these results reveal Runx1 to be key factor whose loss creates ripple effects perturbing the entire transcriptional network (Fig 6).”

9. The discussion is way too long and too speculative for the limited evidence provided.

We shortened the discussion by removing some of the more speculative parts that focused on the possible connection between Runx1 and Zeb proteins in relation to cancer.

Minor Comments

1. Introduction: It would be helpful to describe the differences between Runx1 and Runx2 so the different behaviours discussed later can be better understood.

The major difference between Runx1 and Runx2 has primarily been thought to be different cell-types in which they are expressed to carry out their function. We now briefly describe in the introduction that although highly homologous, Runx proteins do have unique posttranslational modifications that can allow protein interactions that have previously been shown to underly paralog-specific functions.

“Although the Runx proteins often play redundant roles when co-expressed given their homologous protein structures, post-translational modification-dependent co-factor interactions enable unique functions of specific Runx proteins (14-16). (Wang et al., 2011 (PMID 21764752), Goyama et al., 2015 (PMID 25263451), Lee et al., 2019 (PMID 31015486)).”

2. Methods: Some detail is missing from the methods section making the study difficult to interpret and impossible to replicate. There is no information on RNA-seq analysis past alignment - how were differential genes called? Primer sequences and antibodies used are not provided. Data has been aligned to mm9, unless there is a good reason for this mm10 (released 2011) should be used.

We have now included a discussion of the RNA-seq analyses, added the antibody catalog numbers to the methods section and provided the primer sequences as a supplemental Excel file.

All of the data has been mapped to mm9 prior to the downstream analyses so remapping to mm10 would be a substantial undertaking. Instead, we have provided peak bed files that have been ‘lifted over’ to mm10 in the supplement, and also deposited them (along with the mm9 bed files) on the associated GEO page.

3. The authors mentioning the “pioneering” function of RUNX1. However, this idea is way too simplistic. The review they cite does not show such a thing. RUNX1 when expressed can cause the opening of chromatin, but it may need other factors to do this. The authors are advised to read one of Ken Zaret’s reviews for a correct definition of pioneer factors

We agree that we do not have all the evidence to show that Runx1 fully fulfills Zaret’s pioneer function criteria, so we have modified the text to remove mentions of “pioneer function” and now simply describe it as being necessary for maintaining chromatin accessibility, which is better fitting to the data that we provide.

4. Supplementary Figure 3 panels A-C are missing.

Supplementary Fig 3 panels A-C were accidently lost during conversion to pdf and have now been included. Note that they are panels A, C and D now due to the addition of a new panel B.
